# Development and clinical translation of *ex vivo* gene therapy

**DOI:** 10.1016/j.csbj.2022.06.015

**Published:** 2022-06-11

**Authors:** Xiaomo Wu, Xiaorong He, Fahui Liu, Xiaochang Jiang, Ping Wang, Jinyan Zhang, Ju Jiang

**Affiliations:** a Dermatology Institute of Fuzhou, Dermatology Hospital of Fuzhou, Xihong Road 243, Fuzhou 350025, China; b Department of Biomedicine, University of Basel, Klingelbergstr. 70, CH-4056 Basel, Switzerland; c School of Life Sciences, Xiamen University, Xiamen 361005, China; d Department of Medical Biochemistry and Cell Biology, Institute of Biomedicine, University of Gothenburg, 40530 Gothenburg, Sweden; e University of Toronto Scarborough, 1265 Military Trail, M1C-1A4 Ontario, Canada; f Humvira Therapeutics, East Lake High-tech Campus, Wuhan 430074, China; g Department of Dermatology, Juntendo University School of Medicine, 2-1-1 Hongo, Bunkyoku, 113-8421 Tokyo, Japan

**Keywords:** Gene therapy, *ex vivo*, retroviral vectors, stem cells, clinical approvals

## Abstract

Retroviral gene therapy has emerged as a promising therapeutic modality for multiple inherited and acquired human diseases. The capability of delivering curative treatment or mediating therapeutic benefits for a long-term period following a single application fundamentally distinguishes this medical intervention from traditional medicine and various lentiviral/γ-retroviral vector-mediated gene therapy products have been approved for clinical use. Continued advances in retroviral vector engineering, genomic editing, synthetic biology and immunology will broaden the medical applications of gene therapy and improve the efficacy and safety of the treatments based on genetic correction and alteration. This review will summarize the advent and clinical translation of *ex vivo* gene therapy, with the focus on the milestones during the exploitation of genetically engineered hematopoietic stem cells (HSCs) tackling a variety of pathological conditions which led to marketing approval. Finally, current statue and future prospects of gene editing as an alternative therapeutic approach are also discussed.

## Graphical abstract

**Figure:**
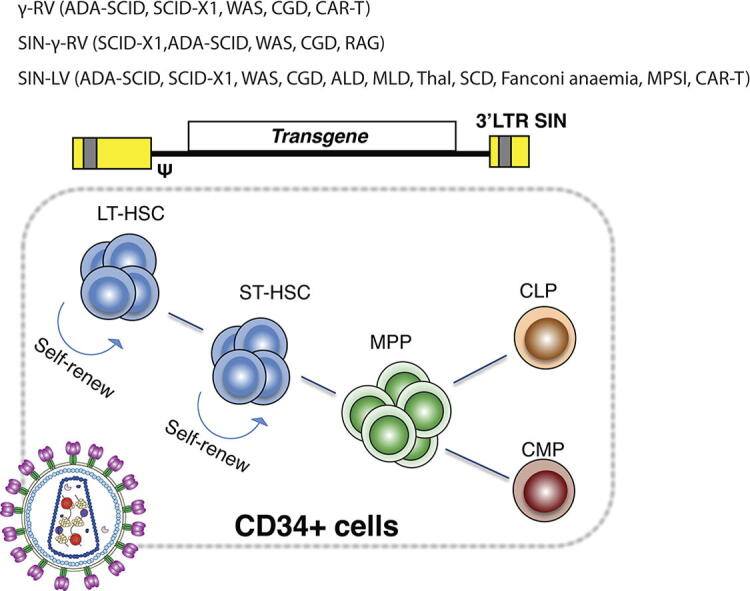

## Abbreviations

RSV: Rous sarcoma virus
HbS: hemoglobin S
CMV: cytomegalovirus
HSCs: hematopoietic stem cells
GT: gene therapy
NIH: National Institutes of Health
EMA: European Medicines Agency
FDA: Food and Drug Administration
γ-RVs: gamma-retroviral vectors
LVs: lentiviral vectors
SIN-LV: self-inactivate lentiviral vector
LTR: long terminal repeat
CAR: chimeric antigen receptor
ADA: adenosine deaminase
SCID: severe combined immunodeficiency
ALL: acute lymphoblastic leukemia
DLBCL: diffuse large B-cell lymphoma
FL: follicular lymphoma
TDT: transfusion dependent β-thalassemia
HBA: hemoglobin subunit alpha
HBB: hemoglobin subunit beta
HBD: hemoglobin subunit delta
HBE: hemoglobin subunit epsilon
HBG: hemoglobin subunit gamma
r/r: relapsed or refractory
MM: multiple myeloma
MCL: mantle cell lymphoma
BCMA: B cell maturation antigen
GFRt: truncated growth factor receptor
AAVs: adeno-associated viruses
Ads: adenoviruses
MoMLV: Moloney murine leukemia virus
allo-BMT: allogeneic bone marrow transplantation
allo-HSCT: allogeneic HSCs transplantation
GvHDs: graft-versus-host diseases
PIDs: primary immunodeficiencies
SCID-X1: X-linked SCID
ADA-SCID: adenosine deaminase deficiency SCID
T-ALL: T-cell acute lymphoblastic leukemia
AEs: adverse events
HLA: human leukocyte antigen
GSK: GlaxoSmithKline
WAS: Wiskott-Aldrich syndrome
CGD: chronic granulomatous disease
IL2RG: interleukin 2 receptor subunit gamma
RAG1: recombination activating gene-1
RAG2: recombination activating gene-2
WASP: Wiskott-Aldrich syndrome protein
ROS: reactive oxygen species
NADPH: nicotinamide adenine dinucleotide phosphate hydrogen
LT-HSCs: long-term hematopoietic stem cells
ST-HSCs: short-term hematopoietic stem cells
MPP: multipotent progenitor
CLP: common lymphoid progenitor
CMP: common myeloid progenitor
MEP: megakaryocytic erythroid progenitor
GMP: granulomonocytic progenitor
BBB: blood brain barrier
RBC: red blood cell
FANC: Fanconi anemia complementation group
CD40LG: CD40 ligand
DCLREIC: DNA cross-link repair 1C
FOXP3: forkhead box protein P3
PRF1: perforin 1
BTK: Bruton tyrosine kinase
F8: coagulation factor VIII
F9: coagulation factor IX
F10: coagulation factor X
PKLR: pyruvate kinase L/R
RPS19: ribosomal protein S19
ELANE: elastase, neutrophil expressed
CYBA: cytochrome B-245 alpha chain
CYBB: cytochrome B-245 beta chain
NCF1: neutrophil cytosolic factor 1
ITGB2: integrin subunit beta 2
ABCD1: ATP binding cassette subfamily D member 1
ARSA: arylsulfatase A
IDS: iduronate 2-sulfatase
IDUA: alpha-L-iduronidase
MPD: methyl parathion degrading
GALC: galactosylceramidase
SGSH: N-sulfoglucosamine sulfohydrolase
NAGLU: N-acetyl-alpha-glucosaminidase
HGSNAT: heparan-alpha-glucosaminide N-acetyltransferase
GNS: glucosamine (N-acetyl)-6-sulfatase
GALNS: galactosamine (N-acetyl)-6-sulfatase
GLB1: beta-galactosidase-1
ARSB: arylsulfatase B
GUSB: glucuronidase beta
ASAH1: N-acylsphingosine amidohydrolase 1
NAGA: alpha-N-acetylgalactosaminidase
GLA: alpha-galactosidase A
GAA: alpha-glucosidase
GBA: glucosylceramidase beta
SCD: sickle cell disease
LCR: locus control region
HSs: hypersensitive sites
Hb: blood hemoglobin
AML: acute myeloid leukemia
MDS: myelodysplastic syndrome
iNMDs: inherited neurometabolic disorders
CNS: central nervous system
LSDs: lysosomal storage disorders
M6P: mannose-6-phosphate
X-ALD: X-linked adrenoleukodystrophy
CALD: cerebral adrenoleukodystrophy
MLD: metachromatic leukodystrophy
MPSI: mucopolysaccharidosis Type I
MPS IIIA: mucopolysaccharidosis Type IIIA
NINDS: National Institute of Neurological Disorders and Stroke
EB: Epidermolysis bullosa
ESCs: epidermal stem cells
MEFs: mouse embryonic fibroblasts
EBS: EB simplex
JEB: junctional EB
DEB: dystrophic EB
KEB: Kindler EB
BMZ: epidermal basement membrane zone
scFv: single-chain variable fragment
MHC: major histocompatibility complex
TCRs: T cell receptors
CLL: chronic lymphocytic leukemia
iPSCs: induced pluripotent stem cells
RCV: replication competent virus
VSV-G: vesicular stomatitis virus glycoprotein
RRE: Rev responsive element
cPPT/CTS: central polypurine tract/central termination sequence
WPRE: woodchuck post-transcriptional regulatory element
ZFNs: zinc-finger nucleases
TALENs: transcription activator-like effector nucleases
CRISPR/Cas: clustered regularly interspaced short palindromic repeats/CRISPR-associated protein
PD-1: programmed cell death protein 1
CISH: cytokine-induced SH2 protein
B2M: β-2 microglobin
DSBs: double-strand breaks
NHEJ: non-homologous end joining
HDR: homology directed repair
NSCLC: non-small cell lung cancer
HPFH: hereditary persistence of fetal hemoglobin
HbF: fetal hemoglobin
HbA: adult hemoglobin
LAM-PCR: linear amplification-mediated PCR

## 1 Introduction

Gene therapy (GT) is the transfer of genetic material to target cells to treat disease [1], [2], through restoration of cell function in inherited abnormalities or rendering cells with new capability in acquired disorders. Dating back to 1953, elucidating the double helix structure of DNA by Watson&Crick [3] profoundly and naturally led to the question regarding the possibility of pinpointing and correcting faulty genes to cure genetic diseases, which underlies the early-stage conceptualization of gene therapy. With rapid growth of recombinant DNA technology during the 1960s [4], [5], the concept and approach of molecular medicine using DNA as therapeutic agent to replace defective genes continued to evolve and became increasingly concrete and attainable [6], [7], [8]. In 1972, T. Friedmann and R. Roblin comprehensively reviewed and addressed the immense potential of GT as well as various key bottlenecks for developing this revolutionary personalized medicine [8]. Translation started in 1990, when the first approved clinical study took place at the US National Institutes of Health (NIH) for a rare immunodeficiency disorder [9]. However, a number of these early clinical studies, including the first NIH trial, could not provide indisputable therapeutic results but exposed unexpected, in several cases devastating, therapy-related toxicities [7], [10]. Fortunately, these setbacks and impediments fueled more basic research and directed the focus on the biology governing the efficacy and safety of gene therapy, ultimately giving rise to successful translation in the 2000s. As a result, multiple GT products obtained approval for marketing (shown in Table 1) and more than 5,000 gene therapy trials worldwide have been registered in clinicaltrials.gov (accessed on 25th May 2022).

**Table 1:** *Ex vivo* gene therapy products that have acquired marketing authorization.

**Product**	**Developer**	**Approval**	**Indication**	**Mechanism of action**	**Clinical trials**	**Price**
STRIMVELIS®	GlaxoSmithKline	May 2016 (EMA)	ADA-SCID	Autologous HSCs expressing *ADA*; γ-RV-*ADA*	NCT00598481; NCT0059978	594,000 $
KYMRIAH® Tisagenlecleucel	Novartis	Aug 2017 (FDA)	B-cell acute lymphoblastic leukemia (ALL), r/r diffuse large B-cell lymphoma (DLBCL)	Autologous CAR T cell targeting CD19; SIN-LV-CAR	NCT02435849; NCT02445248; NCT02228096; NCT02445222; NCT01626495; NCT01029366; NCT01747486; NCT02030847; NCT02030834; NCT02135406	475,000 $
YESCARTA® Axicabtagene Ciloleucel	Kite Pharma (Gilead)	Oct 2017 (FDA)	Non-Hodgkin lymphoma, B-cell lymphoma, high grade B-cell lymphoma and DLBCL arising from follicular lymphoma (FL)	Autologous CAR T cell targeting CD19; γ-RV-CAR	NCT02348216	373,000 $
ZYNTEGLO^TM^ Betibeglogene Autotemcel	Bluebird bio	May 2019 (EMA)	Transfusion dependent β-thalassemia (TDT)	Autologous HSCs expressing *HBB*; SIN-LV-*HBB*	NCT01745120; NCT02151526	∼1.8 million $
LIBMELDY^TM^ Atidarsagene Autotemcel	Orchard Therapeutics	Dec 2020 (EMA)	Early-onset metachromatic leukodystrophy (MLD)	Autologous HSCs expressing *ARSA*; SIN-LV-*ARSA*	Eudract 2009-017349-77 (Study 201222)	∼3.9 million $
BREYANZI® Lisocabtagene Maraleucel	Juno Therapeutics (Bristol-Myers Squibb)	Feb 2021 (FDA)	r/r large B cell lymphoma	Autologous CAR T cell targeting CD19; SIN-LV-CAR*	NCT02631044; NCT03484702; NCT03744676; NCT03310619; NCT03483103; NCT03331198; NCT03743246; NCT03435796	∼ 432,055 $
ABECMA® Idecabtagene Vicleucel	Celgene (Bristol-Myers Squibb)	Mar 2021 (FDA)	r/r multiple myeloma (MM)	Autologous CAR T cell targeting BCMA; SIN-LV-CAR	NCT03361748; NCT03601078; NCT03651128; NCT03435796; NCT02658929; NCT02786511	419,500 $
TECARTUS® Brexucabtagene Autoleucel	Kite Pharma (Gilead)	Oct 2021 (FDA)	r/r mantle cell lymphoma (MCL), r/r B-cell precursor ALL	Autologous CAR T cell targeting CD19; γ-RV-CAR^#^	NCT02601313, NCT02614066, NCT02625480, NCT03624036, NCT04162756	373,000 $
CARVYKTI^TM^ Ciltacabtagene Autoleucel	Janssen Biotech	Feb 2022 (FDA)	r/r MM	Autologous CAR T cell targeting BCMA; SIN-LV-CAR	NCT03548207; NCT04133636; NCT04181827	465,000 $

*The BREYANZI SIN-LV-CAR includes a nonfunctional truncated growth factor receptor (GFRt) that is co-expressed with CD19-specific CAR.

#The γ-RV-CAR applied in TECARTUS is identical to that in YESCARTA, with TECARTUS differing from YESCARTA in that T cell enrichment is performed in TECARTUS manufacture but not in YESCARTA.

γ-RV: Gamma-retroviral vector; SIN-LV: Self-inactivate lentiviral vector; CAR: Chimeric antigen receptor; ADA: Adenosine deaminase; SCID: Severe combined immunodeficiency; r/r: Relapsed or refractory; HBB: Hemoglobin subunit beta; ARSA: Arylsulfatase A; BCMA: B cell maturation antigen.

Treating diseases via gene therapy is achieved through efficient transfer and expression of the therapeutic genetic material, namely nucleic acids DNA or RNA, into the cells of interest. As the plasma membrane or endosome represents impermeable barrier for any large polyanion molecule [11], gene transfer must be conducted either by injection or electroporation (physical method), or facilitated by cationic lipids or polymers coating (chemical method), or utilize biological tools such as viral vectors [11]. Apparently, viral vectors have been chosen in the vast majority of GT-related clinical studies as well as most of the early research and development. Indeed, with the remarkable ability of introducing their genetic material into target cells during the replicative cycle, relying on just few truly simplified protein molecules encoded in the viral genome, viruses are an ideal gene delivery tool of high efficiency. Three viral vector platforms derived from retroviruses, adeno-associated viruses (AAVs) and adenoviruses (Ads) comprise around 80% of all GT applications in development (source: pharmaintelligence.informa.com).

Basically, gene therapy can be broadly divided into two classes: *ex vivo* and *in vivo*. *Ex vivo* gene therapy involves the harvesting of target cells from patient/third party’s body, followed by subsequent viral transduction to genetically modify the harvested cells by introducing functional copies of a desired gene or by gene editing. The transduced cells are then reinfused to the patient to correct the phenotype of the disease. Integrating retrovirus derived vectors are typically involved in the manipulation of hematopoietic or other types of stem cells in *ex vivo* gene therapy [12], [13], [14], [15], [16]. Conversely, AAVs and Ads are more suited for *in vivo* application, where the therapeutic payload is delivered into postmitotic cells and maintained in the cells as episomes [17], [18]. For the last four decades, retrovirus mediated *ex vivo* GT has been a major player in the field of gene therapy [19]. In early 1980s when moloney murine leukemia virus (MoMLV) was initially vectorized, yielded gamma-retroviral vectors (γ-RVs) became the first genetic tool to permit an efficient gene transfer and stable integration in mammalian cells [12], [13], [15], [20], [21]. And in 1991, γ-RV was the first vector used in the NIH clinical trial [9], and a decade later, in the first ever successful clinical study that literally achieved the cure of a genetic disease [22]. In this review, we provide historical and biological perspectives on the advent and clinical implementation of *ex vivo* retroviral gene therapy, focusing on the milestones during the development of HSCs-based GTs and immunotherapies that led to the clinical approvals. In the end, the rapid growth and future prospects of gene editing in the application of *ex vivo* gene therapy are also covered for discussion.

## 2 *Ex vivo* gene therapy

**Fig. 1:**
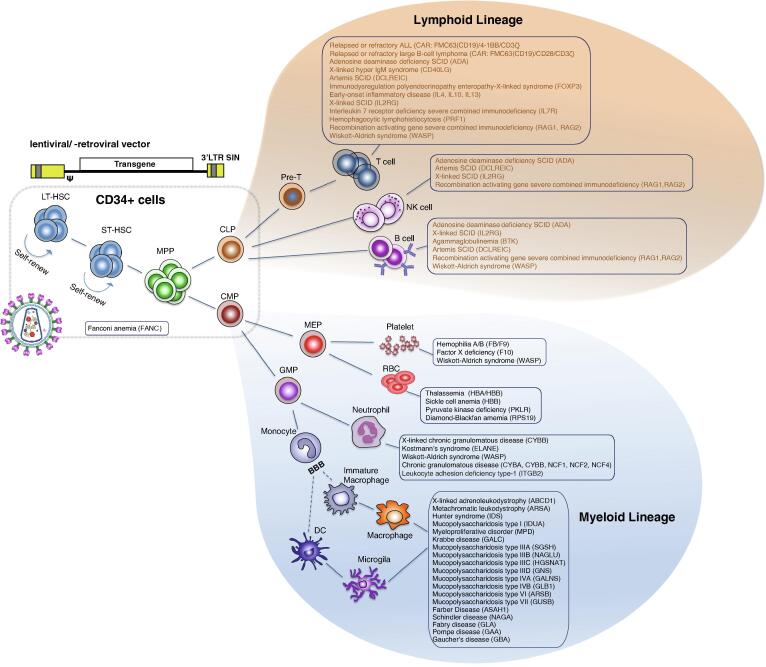
Overview of the hematopoietic hierarchy and genetic disorders potentially curable via *ex vivo* gene therapy. The cell surface marker CD34 is used to enrich a mixture of HSCs for gene therapy. HSCs progressively acquire lineage specifications and differentiate into lineage-committed progenitors and eventually terminally differentiated cells. Examples of *ex vivo* gene therapy under investigation or with translational potential are represented along with affected cell types. LT-HSCs: long-term hematopoietic stem cells; ST-HSCs: short-term hematopoietic stem cells; MPP: multipotent progenitor; CLP: common lymphoid progenitor; CMP: common myeloid progenitor; MEP: megakaryocytic erythroid progenitor; GMP: granulomonocytic progenitor; BBB: blood brain barrier; RBC: red blood cell; FANC: Fanconi anemia complementation group; ALL: acute lymphoblastic leukemia; SCID: severe combined immunodeficiency; ADA: adenosine deaminase; CD40LG: CD40 ligand; DCLREIC: DNA cross-link repair 1C; FOXP3: forkhead box protein P3; IL2RG: interleukin 2 receptor subunit gamma; PRF1: perforin 1; RAG1: recombination activating 1; WASP: Wiskott-Aldrich syndrome protein; BTK: Bruton tyrosine kinase; F8: coagulation factor VIII; F9: coagulation factor IX; F10: coagulation factor X; HBA: hemoglobin subunit alpha; HBB: hemoglobin subunit beta; PKLR: pyruvate kinase L/R; RPS19: ribosomal protein S19; ELANE: elastase, neutrophil expressed; CYBA: cytochrome B-245 alpha chain; CYBB: cytochrome B-245 beta chain; NCF1: neutrophil cytosolic factor 1; ITGB2: integrin subunit beta 2; ABCD1: ATP binding cassette subfamily D member 1; ARSA: arylsulfatase A; IDS: iduronate 2-sulfatase; IDUA: alpha-L-iduronidase; MPD: methyl parathion hydrolase; GALC: galactosylceramidase; SGSH: N-sulfoglucosamine sulfohydrolase; NAGLU: N-acetyl-alpha-glucosaminidase; HGSNAT: heparan-alpha-glucosaminide N-acetyltransferase; GNS: glucosamine (N-acetyl)-6-sulfatase; GALNS: galactosamine (N-acetyl)-6-sulfatase; GLB1: beta-galactosidase-1; ARSB: arylsulfatase B; GUSB: glucuronidase beta; ASAH1: N-acylsphingosine amidohydrolase 1; NAGA: alpha-N-acetylgalactosaminidase; GLA: α-galactosidase A; GAA: alpha-glucosidase; GBA: glucosylceramidase beta. (For interpretation of the references to colour in this figure legend, the reader is referred to the web version of this article.)

Largely due to decades of clinical management of diverse blood disorders and hematologic malignancies by applying allogeneic bone marrow transplantation (allo-BMT), the population of HSCs was chosen as the first and primary target for *ex vivo* gene therapy [23]. After eradication of abnormal blood/immune cells, HSCs transplantation is undertaken to reconstitute the blood/immune system and subsequently establish sustainable hematopoiesis. However, despite allogeneic HSCs transplantation (allo-HSCT) being implemented as a standard treatment [24], engineered autologous transplantation potentially exhibits multiple advantages over allogeneic approach [25], [26], such as requiring no histocompatible donor, eliminating the complications of graft-versus-host diseases (GvHDs), avoiding immunosuppressant lifelong administration, among others. Therefore, genetically modifying autologous HSCs appears to be the most promising application in the field of gene therapy tackling hematologic disorders and immune aberrations (Fig. 1).

### 2.1 Severe combined immunodeficiency (SCID)

SCID is the most severe form of primary immunodeficiencies (PIDs), with an estimated incidence between 1:40,000 to 1:75,000 live births [27], [28]. Initially known as swiss-type agammaglobulinemia, SCID syndromes are characterized by the profound deficiency of T cells combined with varied abnormalities in B and NK cells. More than 20 different genes have been implicated in SCIDs [27] and the most common types are X-linked SCID (SCID-X1) and adenosine deaminase deficiency SCID (ADA-SCID), accounting for 30–40% and 15%-20% of all diagnosed [28], [29], [30], respectively. Therapeutic interventions consist of allo-HSCT [31], [32], enzyme replacement therapy and theoretically, gene therapy to correct or alleviate underlying genetic defects.

In the history of gene therapy, pioneer clinical trials were predominately driven by the goal to treat life-threatening SCIDs, counting on the vectorization of MoMLV to generate functional vehicles for gene transfer (1st generation γ-RVs). In 1990, the very first officially authorized gene therapy trial to restore ADA deficiency was conducted at the NIH, in which autologous T cells were collected from peripheral blood and transduced by γ-RV carrying human 1.5 kb *ADA* cDNA [9], [33], [34]. Although prolonged survival of genetically modified T lymphocytes had been observed after a decade [35], it is not possible to determine the clinical benefit from this treatment of *ADA* gene augmentation GT due to in parallel deployed enzyme replacement therapy.

Later on, in the late 1990s, a clinical study conducted by M. Cavazzana-Calvo and colleagues from Necker Hospital in Paris [22], [36] and Great Ormond Street Hospital in London [37] illustrated convincing therapeutic effects of gene therapy treating SCID-X1, representing the first clear GT success in human trial [23], [25], [38]. SCID-X1 is caused by loss-of-function mutations in the gene encoding the γc subunit of cytokine receptors like IL-2, -4, -7, -9, -15 and IL-21, causing the depletion of circulating T and NK cells and poorly performing B lymphocytes [29]. In these SCID-X1 trials, CD34^+^ cells were purified from bone marrow from 20 patients and transduced by γ-retroviruses with the expression cassette of *IL2RG* gene encoding γc subunit to reconstitute a range of cytokine receptors affected. Long-term immune correction and restoration of the primary immunodeficiency have been observed in most of the treated children, with substantial T-cell recovery within 2–5 months for 19/20 patients, including the capabilities of clearing of SCID-related infections, responding to standard childhood vaccinations and gain in growth [37], [39], [40], [41]. Unfortunately, T-cell acute lymphoblastic leukemia (T-ALL) developed in 6 patients 2–14 years after the treatment and one of whom did not respond well to chemotherapy and died [39], [40], [41]. In-depth analysis revealed T-ALLs were caused by insertional mutagenesis, resulted from provoking unconstrained activation of proto-oncogenes (e.g., *LMO-2*, *CCND2*, *MECOM*) due to integrated strong intact viral enhancer/promoter of γ-RV in proximity [42], [43], [44].

Thus, collective efforts have been accordingly devoted to improving the first generation γ-RVs to minimize the risk of adverse events (AEs), mainly through the deletion of the viral enhancer/promoter elements of the 3′-LTR to attain self-inactivate (SIN) γ-RVs and driving the expression of *IL2RG* transgene by other promoters, such as EF1a instead [45]. New generation of vector was tested in multiple phase I/II trials for the infant patients with confirmed mutation in *IL2RG* gene [45], [46], [47] and lacking HLA-identical donor or being refractory to standard therapy. The data published in a preliminary report in 2014 (NCT01410019, NCT01175239, and NCT01129544) [45] indicated comparable recoveries between the previous clinical trials applying 1st generation γ-RV and the ongoing trials adopting SIN-γ-RV system. So far, no leukemia in any of the patients has been reported, suggesting encouraging improvement in the safety as a result of the SIN-principle implementation [48], [49], [50], [51]. Meantime, during the period of γ-RVs system advancing and evolving, the SIN concept has also been introduced to improve the safety of lentiviral vectors (LVs), another retroviral vector platform that generally exhibiting safer integration pattern as well as better transduction efficiency in quiescent HSCs [52], [53], [54], [55], [56]. Very recently, multiple trials based on SIN-LVs for SCID-X1 gene therapy have been conducted and to date, no oncogenesis has been reported, albeit with relatively short follow-up [29], [39], [48], [57], [58], [59], [60].

Further on, for ADA-SCID, although the initial study failed to generate unambiguous beneficial results as described above, irrefutable success was achieved in the clinical trials performed at San Raffaele Hospital in Milan and Hadassah Hospital in Jerusalem in the early 2000s [61]. Combining the utilization of busulfan to promote the cellular engraftment and the discontinuation of enzyme replacement treatment for endowing survival advantage to genetically modified cells, autologous *ex vivo* gene therapy was demonstrated to be a safe, effective and durable intervention for SCID patients with ADA deficiency [62]. Notably, dissecting integration profiles found little difference between γ-RVs mediated *ADA* insertion and that of *IL2RG*, but the insertional mutagenesis that overshadowing the SCID-X1 trials were never observed in the ADA-trials, implying apart from LTR, the transgene product itself could involve in or even contribute to carcinogenesis [63]. In 2016, the treatment of *ADA* gene transfer mediated by γ-RV received EMA approval and became the first marketed *ex vivo* gene therapy in Europe [64], [65]. The product, Strimvelis® (GSK2696273), has been commercially manufactured by the GlaxoSmithKline (GSK) based in Raffaele Hospital [32], [64]. Furthermore, excellent results have been reported lately with SIN-lentiviral based approach for ADA-SCID treatment (NCT01852071, NCT02999984, and NCT01380990) [24], [66], showing robust immune reconstitution, sustained metabolic detoxification and recovered ADA activity level.

### 2.2 Other PIDs

PIDs are a heterogeneous group of more than 450 rare, chronic conditions associated with impaired immune development and function, globally affecting 1:10,000 live births [67], [68], [69], [70], [71]. More than 260 genes have been elucidated underlying the pathogenesis of PIDs and new genetic defects are continually identified because of improved diagnostic capability based on next-generation sequencing technologies [72]. Similar to SCIDs, other types of PIDs that can be treated by allo-HSCT are in principle eligible for autologous HSCs gene therapy [67], [68], [70], [72], such as Wiskott-Aldrich syndrome (WAS), chronic granulomatous disease (CGD), recombination activating gene severe combined immunodeficiency (RAG1, RAG2) and so on. For instance, WAS is a rare X-linked recessive PID disorder characterized by recurrent infections and elevated manifestations of autoimmune diseases and tumors. It caused by the mutations in the gene *WAS* encoding Wiskott-Aldrich syndrome protein (WASP) [73], [74], [75]. The first attempt to treat WAS by gene therapy was done in Germany in 2006 [75], [76]. Sustained and therapeutic WASP expression delivered by γ-RV system was achieved in transduced HSCs, myeloid and lymphoid cells, and platelets after transplantation, however, unacceptable leukaemic toxicity occurred. Recently, adopting more advanced vehicle platform showed some encouraging progresses in multiple clinical studies [73], [74], [77], [78]. Of note, SIN lentiviral vector incorporating a 1.6 kb fragment from the promoter region of *WAS* gene has been proved capable of mediating considerably high WASP expression, and abrogation of bleeding and sustained immunofunction were achieved with no clonal imbalance and dysregulation detected [21], [79], [80].

CGD is an another type of PID disorder, which affecting the ability of phagocytes to destruct ingested pathogens due to the incapability to generate reactive oxygen species (ROS) for germ killing [81], [82], [83], [84]. Patients with CGDs are highly susceptible to frequent and sometimes lethal opportunistic bacterial and fungal infections. For ROS production, phagocyte NADPH oxidase is first assembled by three cytosolic phox subunits, p47^phox^, p67^phox^ and p40^phox^, and then translocated to the membrane-bound flavocytochrome, consisting of subunits gp91^phox^ and p22^phox^. The most predominant form of CGDs is an X-linked condition (X-CGD) caused by the mutations in *CYBB*, the gene encoding the subunit gp91^phox^, and the rest forms of CGDs are autosomal recessive, from the mutations in *CYBA*, *NCF1*, *NCF2* and *NCF4*, which encode p22^phox^, p47^phox^, p67^phox^ and p40^phox^, respectively. Early *ex vivo* GT clinical trials applying γ-RVs to correct defective gp91^phox^ and p47^phox^ were mostly disappointing due to the lack of therapeutic efficacy [85], [86], [87] for various reasons. In the early trial studies that were initiated in the mid-1990s, bone marrow conditioning or myelosuppression was not performed prior to transplantation to make space for the genetically modified cells to engraft. Then later, other factors undermining the efficacy of GT treatment have been revealed. Persistent chronic inflammation typically associated with CGD causes the viral promoter methylation, leading to transgene expression silencing [88]. In addition, the hematopoietic proliferative stress induced by chronic inflammation reduces the fitness of harvested HSCs [89], further lowering viral transduction efficiency and engraftment. Moreover, some trials based on γ-RVs delivery system were complicated by the development of myelodysplasia and leukemia-like growth of blood cells [84], [85], [88], [90]. Notably, however, introducing SIN-LV system with some added regulatory elements resistant to epigenetic modification was recently exploited, which has shown some promising therapeutic results in several patients with severe X-linked CGDs (NCT02234934, NCT01855685) [91].

In short, the multiple clinical trials for treating SCIDs demonstrated the curative potential of gene therapy and the lesson learned from tackling SCIDs not only provided valuable guidance but also generated momentum for pursuing the translational research for a variety of PIDs [23], [68], [70], [71], [92], [93]. The properties of multipotency and readiness for *in vitro* manipulation and repopulation have placed HSCs at the frontline of genetically engineering *ex vivo* therapy (indicated in Fig. 1).

### 2.3 β-Hemoglobinopathies

Sickle cell anemia (also known as sickle cell disease, SCD) and β-thalassemia are two of the most common monogenic diseases [94], both caused by the mutation in the gene encoding the adult β-globin chain of hemoglobin (*HBB,* hemoglobin subunit beta), with high prevalence primarily found in South Asia, Middle East, Mediterranean and North Africa [95], [96], [97], [98]. SCD is characterized by a single mutation that leads to negatively charged glutamic acid replaced by hydrophobic valine [99], producing defective peptide of hemoglobin S (HbS) prone to polymerization. As a result, it causes erythrocyte distortion, hemolysis, anemia, vaso-occlusive episodes and irreversible endoorgan damage [100]. In contrast, almost 300 pathological mutations have been identified underlying β-thalassemia etiology (http://globin.cse.psu.edu), which impede the transcription, RNA processing, or translation of *HBB* [101], [102], [103]. The clinical presentation of β-thalassaemia varies from being mild asymptomatic to seriously severe disease, and for the latter ineffective erythropoiesis may require constant blood transfusion and iron chelation to sustain life.

**Fig. 2:**
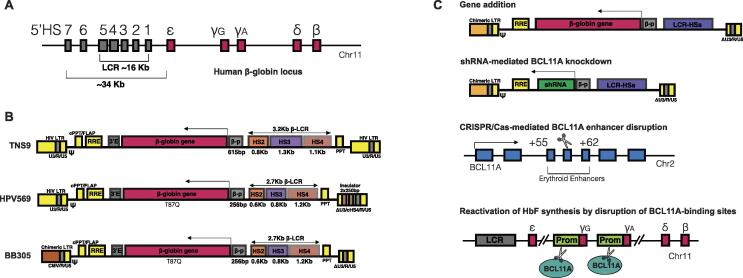
Overview of human β-globin locus and *ex vivo* gene therapy for the treatment of SCD and TDT. A) Schematic representation of human β-globin locus and LCR; B) Erythroid-specific expression of β-like globin transgenes using lentiviral vectors; C) Schematic representation of lentiviral gene addition therapy and HBB mutation-independent therapeutic approaches for the treatment of SCD and TDT.

Allo-HSCT can be curative for β-hemoglobinopathies, however, less than 20% of eligible patients have HLA-matched donors [100], [102]. Being the most prevalent inherited diseases worldwide [104], both SCD and β-thalassemia have been a key focus of gene therapy to attain corrected autologous HSCs. However, the main clinical challenge to tackle β-hemoglobin aberration genetically is to express functional protein in erythroid specific lineage and in substantial amounts to reach therapeutic benefits [101]. Early studies on the ectopic expression of β-globin gene (*HBB*) as well as some forms of β-thalassemia led to the discovery of the locus control region (LCR) that is critical for mediating erythroid lineage specific and genomic position-independent *HBB* expression [105], [106], [107], [108], [109], [110]. Human β-globin locus, approximately 70 kb in length, comprises five β-like globin genes, linearly arranged in the order they are expressed during development: ε (*HBE1*), γG (*HBG2*), γA (*HBG1*), δ (*HBD*), and β (*HBB*) on chromosome 11 (illustrated in Fig. 2A) [105], [111], [112], [113]. The 5′ regulatory region of β-globin gene cluster spanning 34 kb, upstream to the first embryonic globin gene *HBE1*, includes several DNase I-hypersensitive sites (HSs; 5′HS1-HS7 in the entire region and 5′HS1-HS5 in the LCR), each 200 to 400 bp in size and separated from each other by several kb of DNA [108], [111]. As the LCR is the major element controlling expression of the downstream β-globin genes, laboratories around the world [113], [114], [115], [116] have aimed to reduce the size of LCR while maintaining most of its cis-regulatory function to confer the physiological expression of *HBB* to genetically modified cells [108], [112], [117], [118].

In 2000, a lentiviral vector referred as TNS9 comprising mini LCR elements (840 bp for HS2, 1308 bp for HS3, and 1069 bp for HS4, in total about 3.2 kb in length, shown in Fig. 2B) was demonstrated by May and colleagues capable of effectively correcting thalassemia intermedia in mouse model [108]. In 2010, the clinical trial of LG001 study for the patients with β-thalassaemia showed one of three subjects treated by lentiviral vector HPV569 derived from TNS9 became transfusion-independent 1 year after cell infusion and the blood hemoglobin (Hb) level remained stable around 8.5 g/L for more than 8 years [119]. This vector HPV569 bears “antisickling” β^T87Q^ amino acid substitution and various minor modifications on LCR components (646 bp for HS2, 845 bp for HS3, and 1153 bp for HS4, 2.7 kb in length) [119], [120]. Although the therapeutic benefit was found resulted from a dominant clone with lentiviral insertion occurred inside the *HMGA2* gene [120], the LG001 study provided the proof of principle of tackling β-thalassaemia via engraftment of genetically modified autologous HSCs and paved a path for vector improvement and global phase I/II trials.

Further on, to increase the titre and transduction efficiency of vector HPV569, the strong and constitute CMV promoter was introduced to replace the wild-type U3 within the 5′LTR and two chromatin insulators were thereby removed, yielding SIN-LentiGlobin BB305 vector [121]. In following two phase II trials (HGB-204/NCT01745120 and HGB-205/NCT02151526), the autologous CD34^+^ cells from 22 patients with transfusion-dependent β-thalassemia (TDT) were transduced with BB305 vector and almost all the patients achieved sustained transfusion independence after treatment. In nine patients with the most severe form of the disease, the need for blood transfusions was dramatically reduce by 73% and completely eliminated in three patients [96], [102]. Based on the results of these two clinical trials, LentiGlobin BB305, commercially named Zynteglo (betibeglogene autotemcel), was granted conditional marketing authorisation by EMA in May 2019, for patients 12 years and above with TDT who are eligible for stem cell transplantation but do not have a matching related donor.

In comparison, the BB305 treatment for SCD subjects exhibited varied therapeutic efficacies, presumably due to the hypoxic condition, chronic inflammation, oxidative stress and dysregulated metabolism linked to SCD pathophysiology [122]. In the HGB-205 study, the treatment with BB305 vector resulted in transfusion independence, sustained clinical remission and stabilization of Hb^T87Q^ expression (contributing 47.9% and 25.8% of total Hb, respectively) in two of three patients with SCD [52], [123], [124]. However, the same treatment only produced mild improvement for the third SCD patient, with slightly reduced frequency of transfusion [52]. In the subsequent multicenter HGB-206 study (NCT02140554), the Hb^T87Q^-globin levels in the first few SCD patients after the treatment with lovotibeglogene autotemcel (lovo-cel; formerly as BB305/LentiGlobin bb1111) were low, incapable of preventing SCD-related clinical events and delivering the discontinuation of transfusion [125]. Then, in two additional study arms (group B and C), refined transduction and transfusion protocols, optimized busulfan conditioning and plerixafor mobilization of HSCs, among others, were conducted accordingly, which resulted in complete resolution of severe vaso-occlusive episodes and sustained production of Hb^T87Q^ in the last group C [126]. Further on, in February 2021 two participants in HGB-206 trial were diagnosed with acute myeloid leukemia (AML) and myelodysplastic syndrome (MDS), respectively, which raised concerns regarding lovotibeglogene autotemcel and led to the temporary suspension of HGB-206 and HGB-210 trials and additionally the marketing of betibeglogene autotemcel. The suspension was lifted by FDA several months later after further assessment showed that the trial participant diagnosed with MDS had actually developed transfusion-dependent anemia and the occurrence of AML was unlikely associated with BB305 vector [127].

Overall, the progressive understanding of the molecular, cellular and developmental processes underlying hemoglobin biology and pathology facilitates the optimization of conventional addition GT as well as lays a foundation to test various state-of-art biotechnologies, with high translational potential [96]. For instance, to improve titer and overcome the challenge of sub-optimal gene transfer efficiency to HSCs for the treatment of SCD, considerable efforts have been put on vector enhancement and redesigning, such as reducing LCR length or using “forward-oriented” lentiviral vector [128]. Very recently, Morgan *et al.* managed to substantially shorten LCR cis-regulatory component (∼1.2 kb) through identifying the core enhancer sequences of LCR 5’HS2-HS4 based on genomic and epigenomic analysis, generating a novel β-globin lentiviral vector with high titer and superior transduction performance [129]. In the last section of this review, HBB mutation-independent strategies based on DNA editing or RNA interference for the treatment of β-hemoglobinopathies will be discussed.

### 2.4 Inherited neurometabolic disorders (iNMDs)

The central nervous system (CNS) is more frequently and severely affected by the genetic abnormalities with the enzymes mediating metabolic pathways, such as lysosomal or peroxisomal enzymes [130], [131], [132], than any other organ. Build-up of toxic substrates in the CNS impairs neurodevelopment or causes neurodegeneration, which cannot be easily addressed by enzyme replacement therapy due to the obstacle of the blood–brain barrier (BBB) [133]. For over 20 years, allo-HSCT has been performed to treat such enzyme deficiency diseases as metabolically competent myeloid cells are found capable to infiltrate into the CNS and replace the resident macrophages and microglias to release enzymes (indicated in Fig. 1) [133], [134], [135], [136], [137]. In the case of lysosomal storage disorders (LSDs), once produced and released by migrated progenitor cells of transplanted allogeneic HSCs, lysosomal enzymes can be specifically recognized and bound by extracellular mannose-6-phosphate (M6P) receptors of neighboring cells to ameliorate the neuropathological severity, a mechanism known as cross-correction. Further on, it has been shown 10–15% of residual enzymatic activity is the critical threshold for the manifestations of a range of lysosomal enzyme deficiencies, indicating only a small percentage of correction is required for the physiological turnover and lysosome homeostasis [138], [139], [140]. Even so, some patients still suffer from insufficient enzymatic activity and experience neurological burden despite having successful allo-HSCT. Recent studies showed genetic engineered autologous HSCs modified by γ-RVs or LVs could substantially increase therapeutic enzyme level in the brain after engraftment and enable enhanced cross-correction effects, alleviating inflammation and oxidative stress [137], [141], [142].

For example, metachromatic leukodystrophy (MLD) is caused by the deficiency of lysosomal enzyme arylsulfatase A (ARSA), which leads to the accumulation of sphingolipid sulfatide in the oligodendrocytes, causing oligodendrocytal degeneration and progressive demyelination [140]. Previously, the efficacies of intracerebral GT with AAVs, enzyme replacement therapy and allo-HSCT have been investigated and shown could not effectively reverse the progression of MLD in clinical studies [136], [143], [144], [145], [146], [147]. In contrast, the result of a phase I/II trial (OTL-200/NCT01560182) published at the beginning of this year showed the treatment with arsa-cel (atidarsagene autotemcel, Libmeldy^TM^,Orchard, shown in Table 1), an *ex vivo* gene therapy containing autologous HSCs transduced with SIN-lentiviral vector encoding human *ARSA* cDNA, resulted in sustained, clinically relevant benefits in children with early-onset MLD [148].

Indeed, inherited neurometabolic disorders are individually rare, but they have a collective frequency of ∼1 in 1000 live births (∼1 in 5000 for LSDs alone) [140], [149], making them a significant public health problem worldwide [150], [151], [152]. To date, mutations in almost 700 different proteins were identified causing iNMDs (more than 70 genes for LSDs alone) [153], [154], [155], [156] and this list continues to grow. In Table 2, current *ex vivo* gene therapies tackling various genetic neurometabolic conditions that have entered clinical trials are summarized.

**Table 2:** *Ex vivo* gene therapies for inherited neurometabolic disorders.

**Conditions**	**Clinical trial**	**Interventions**	**Sponsor/Collaborators**	**Phase**	**Enrollment**	**Status**
X-ALD/CALD	NCT02559830	Autologous HSCs transduced with lenti-*ABCD1*	Shenzhen Second People's Hospital	I/II	50	Recruiting
	NCT03852498	Lenti-D^TM*^	Bluebird bio	III	35	Active, not recruiting
	NCT01896102	Lenti-D^TM^	Bluebird bio	II/III	32	Completed
MLD	NCT01560182	OTL-200	Orchard Therapeutics|	I/II	20	Active, not recruiting
	NCT03725670	Autologous HSCs transduced with lenti-*ARSA*	Shenzhen Geno-Immune Medical Institute	Not Applicable	10	Unknown status
	NCT02559830	Autologous HSCs transduced with lenti-*ARSA*	Shenzhen Second People's Hospital	I/II	50	Recruiting
	NCT04283227	OTL-200	Orchard Therapeutics	III	6	Recruiting
	NCT03392987	OTL-200	Orchard Therapeutics	II	10	Active, not recruiting
MPSI	NCT03488394	OTL-203	Orchard Therapeutics	I/II	8	Active, not recruiting
MPS IIIA	NCT04201405	Autologous HSCs transduced lenti-*SGSH*	University of Manchester| Orchard Therapeutics	I/II	5	Active, not recruiting
Fabry disease	NCT03454893	AVR-RD-01	AVROBIO	I/II	11	Terminated
	NCT02800070	Autologous HSCs transduced with lenti-*GLA*	University Health Network, Toronto|Ozmosis	I	5	Active, not recruiting
Gaucher's disease	NCT00001234	Autologous HSCs transduced with γ-retro-*GBA*	NINDS|NIH-Clinical Center	I	120	Completed
	NCT04145037	AVR-RD-02	AVROBIO	I/II	16	Recruiting

*Breakthrough Therapy Designation granted by FDA in May 2018.

X-ALD: X-linked adrenoleukodystrophy; CALD: cerebral adrenoleukodystrophy; ABCD1: ATP binding cassette subfamily D member 1; Lenti-D^TM:^: Autologous HSCs transduced with lenti-*ABCD1*; MLD: metachromatic leukodystrophy; ARSA: arylsulfatase A; OTL-200: Autologous HSCs transduced with lenti-*ARSA*; MPSI: mucopolysaccharidosis Type I; OTL-203: Autologous HSCs transduced with lenti-IDUA; IDUA: alpha-L-iduronidase; MPS IIIA: mucopolysaccharidosis Type IIIA; SGSH: N-sulfoglucosamine sulfohydrolase; GLA: Alpha-galactosidase A; AVR-RD-01: Autologous HSCs transduced with lenti-*GLA*; GBA: glucosylceramidase beta; AVR-RD-02: Autologous HSCs transduced with lenti-*GBA*; NINDS: National Institute of Neurological Disorders and Stroke.

### 2.5 Epidermolysis bullosa (EB)

Skin can be an ideal and appealing target for *ex vivo* gene therapy as the epidermal stem cells (ESCs) contained in the basal layer and hair follicles sustain the continuous renewal of epidermis, allowing the ESCs within epidermis to be easily harvested and subjected to *ex vivo* manipulation [157]. On the other hand, the advancement of *in vitro* cultivation of human skin cells by the co-culture with 3T3 mouse embryonic fibroblasts (MEFs) as feeder cells provided long-term expansion solution of human primary epidermal keratinocytes without immortalization, which made ESCs based *ex vivo* intervention to restore severe skin, mucosal and corneal defects realistic [157], [158]. Undoubtedly, applying genetically corrected ESCs to treat some rare hereditary skin diseases represented some of those unprecedented achievements in the whole field of gene therapy [159], [160], [161], [162], [163].

EB is a group of rare genetic skin fragility disorders characterized by mucocutaneous blister formation in response to minimal mechanical trauma due to reduced skin resilience [164], [165], [166]. Depending on the location of the molecular defects within the skin, EB can be divided into four major types, EB simplex (EBS), junctional EB (JEB), dystrophic EB (DEB) and Kindler EB (KEB). At least 16 distinct genes that encoding the structural components critically involved in maintaining the integrity and functionality of epidermis or the dermo-epidermal basement membrane zone (BMZ) have been identified underlying the causes of EBs [160], creating highly variable clinical manifestations ranging from mild localized blistering to severe and generalized erosions.

The first *ex vivo* gene therapy for treating EB was reported by Mavilio, de Luca and colleagues in 2006, tackling junctional subtype [159]. This landmark study demonstrated the efficacy and safety to treat an adult patient with JEB by transplantation of genetically modified epidermal graft sheet, generated from autologous keratinocytes transduced with γ-RV expressing full-length, wild-type *LAMB3* cDNA. The transgenic skin has been maintained and no AEs are noted in the following 16 years follow-up [163]. And more recently, this genetic intervention has achieved the successful replacement of 80% of the body surface area for a 7-year-old patient in 2015, who had a severe form of JEB and life-threatening infection by the time of admission [158]. In particular, two years after the *ex vivo* gene therapy, analyzing the proviral integration sites of γ-RVs to track the clonal expansion of transduced cells verified the ESCs with holoclone-forming capability had been successfully targeted and genetically corrected, providing sustainable therapeutic benefits for the maintenance of epithelial integrity and function [158], [167].

As noted by some mechanistic studies, restoring mechano-signaling upon reintroduction of functional laminin-332 imposes survival and replicative advantages to genetically modified clones, presumably through rectifying YAP/TAZ signaling pathway [161], [168]. Therefore, although encouraging clinical outcomes obtained in the clinical trials with *LAMB3*-deficiency JEB, *ex vivo* gene addition therapy for other types of EBs remain to be greatly challenging. For instance, improved wound healing and restoration of anchoring fibrils at the BMZ were initially observed in the RDEB patients treated with regenerated epidermal sheets overexpressing *COL7A1*, however, type VII collagen level was found significantly decreased at the graft sites within one year [169]. Lately, an innovative strategy was deployed in GENEGRAFT project (NCT01874769; NCT04186650) [170] to transduce both autologous keratinocytes and fibroblasts using SIN-γ-RV-*COL7A1*, aiming to optimize anchoring fibril assembling condition. So far, one patient was treated in the early of 2021 [160].

### 2.6 Chimeric antigen receptors T cells (CAR-T)

In contrast to the approach tackling monogenic diseases, which is mostly straightforward, gene therapy for the treatment of malignment conditions needs to explore and evaluate a wide range of strategies to ensure therapeutic benefits and efficacy, such as locally delivering suicidal genes like *p53* or *TNF-α* to elicit programmed tumor cell death, applying genetically modified oncolytic viruses to enable selective replication in cancerous cells, deploying genetically engineered immune cells to elevate antigen-mediated anti-tumor cytotoxicity, and so forth. Among them, CAR-T therapy emerged as unprecedented revolutionary therapeutics for its way of engaging the immune system in the fight against cancer [171], [172], [173], [174]. Retroviral vector mediated CAR expression renders modified T lymphocytes with novel specificity, function, and metabolism. A CAR consists of a single-chain variable fragment (scFv), derived from antibody and accordingly capable of mediating non-MHC restricted surface antigen recognition, an intracellular signaling domain responsible for T cell activation (typically derived from CD3ζ), and one or more intracellular costimulatory domains to enhance T cell function and persistence (derived from either CD28 or 4‐1BB) [175]. Unlike the physiological T cell receptors (TCRs) binding to antigen, this CAR-architecture overcomes the requirement for HLA recognition and CARs can be engineered to recognize a variety of substances, substantially expanding the repertoire of T cells [176], [177].

Early design of the first generation CARs was based on the observation originated from the cloning of CD3ζ chain, which was found capable of activating T cells independently [178]. For this reason, the 1st generation CARs comprised merely the cytoplasmic CD3ζ segment of endogenous TCR as signal transmitter, without any costimulatory component. Despite some encouraging outcomes in pre‐clinical study, the 1st generation CAR-T cells exhibited limited expansion capacity as well as insufficient cytokine production, failing to induce meaningful anti-tumor effects in the following clinical studies for leukemia, lymphoma and other types of cancers [179]. Fortunately, breakthrough came after the engineering of the 2nd generation CARs by incorporating costimulatory endodomain from either CD28 or 4‐1BB to potentiate the activation, cytotoxicity and persistence of T cells. Albeit with different pharmacokinetics, the 2nd generation CARs-T therapies exhibited significantly stronger antitumor activity [180], [181], [182], [183], [184]. Specifically, CD28/CD3ζ induces prompt and intense signal, whereas 4-1BB/CD3ζ elicits more durable cytotoxic activity with improved persistence *in vivo.*

In 2010, National Cancer Institute reported the first clinical trail showing positive response which mediated by γ-retroviral FMC63(CD19)/CD28/CD3ζ CAR-T cells in a patient with advanced follicular lymphoma (FL) [185]. Shortly after that, another clinical trial on chronic lymphocytic leukemia (CLL) demonstrated durable and effective responses to lentiviral FMC63(CD19)/4-1BB/CD3ζ CAR-T treatment, reported by the University of Pennsylvania, Philadelphia [186]. And more early-phase studies from multiple institutions confirmed the persuasive anti-tumor efficacy in those heavily pretreated patients with B-cell malignancies. Hence, in less than 10 years, CAR-T based *ex vivo* gene therapy has transitioned from preclinical proof-of-concept to FDA approved therapies (demonstrated in Table 1) [187], [188], [189], [190], [191], [192], [193]. In August 2017, the FDA announced the approval of SIN-lentiviral FMC63(CD19)/4-1BB/CD3ζ CAR-T tisagenlecleucel (tisa-cel, KYMRIAH®, Novartis) for the treatment of pediatric patients and young adults with relapsed or refractory ALL. Approval was based on the results of the open-label, multicenter, single-arm Phase II ELIANA trial (CCTL019-B2202), showing 83% of patients (52 out of 63) who received treatment with tisa-cel achieved complete remission (CR). Soon after, in October 2017, γ-retroviral FMC63(CD19)/CD28/CD3ζ axicabtagene ciloleucel (axi-cel, YESCARTA®, Kite Pharma Inc.) was approved for the treatment of adult patients with relapsed or refractory large B-cell lymphoma after two or more lines of systemic therapy, based on the results from multicenter single-armed phase I/II ZUMA-1 trial [180], [181].

CAR-T cells presents an entirely new paradigm in drug development, utilizing artificial receptor to mediate therapeutic benefits for months or years following a single medical intervention. However, cytokine release syndrome, neurotoxicity, relapse due to the loss of the antigen on tumor cells and others remain to be addressed. Investigators around world currently venture into formulating next-generation CAR therapies by targeting different and/or multiple antigens, incorporating additional synergistic components, or testing alternative cell sources, such as allogeneic CAR-T cells, NK cells, or even the T/NK cells obtained from induced pluripotent stem cells (iPSCs). Furthermore, there are numerous ongoing trials exploring the potential of CAR-T therapy in other malignant disorders, including myeloma and solid tumors, which have been systemically summarized in multiple reviews [174], [175], [194], [195], [196] and are not discussed in detail here.

## 3 Integrating retroviral vectors

Retroviruses are enveloped integrative RNA viruses belonging to the Retroviridae family that consists of seven genera [197]. The retroviral particle contains two copies of single-stranded positive-sense molecule, 7–11 kb in length, present in a dimeric form, capable of causing disease in humans and animals. Based on splicing patterns and the presence of auxiliary proteins, retroviruses are classed as simple genera of Retroviridae (i.e., α-, β- and γ-retrovirus), and complex genera (i.e., δ- and ε-retrovirus, lenti-and spumavirus). All retroviruses harbor the minimum three essential genes *gag*, *pol* and *env*, and the complex retroviruses have additional accessory genes, such as *nef*, *vif*, *vpu*, and *vpr*, and regulatory genes such as *tat* and *rev*, to exert control over the virus life cycle and pathogenicity. As a result, simple γ-retrovirus gain access to host genomic DNA only after the nuclear envelope is dissolved during cell division, whereas complex lentiviruses can access host genome via active transportation through the nuclear pore.

The property of genomic integration in the infected cell has made retroviruses a valuable tool to mediate *ex vivo* genetic manipulation when the stable expression of transgene or the transgene inherited by the progeny of the transduced cells are preferred. The γ-RVs developed during the 1980s were the first delivery platform demonstrated with the capability of transferring exogenous gene into repopulating HSCs and thereby have been employed in almost all early clinical trials [1], [13], [20], [198], [199], [200], [201], [202], [203], [204]. However, γ-RVs system developed from simple retrovirus had an inherent limitation due to the lack of capability to infect non-dividing cells. Consequently, LVs derived from complex lentiviruse were added into the vehicles for gene therapy and have becoming the predominantly used retroviral carrier system [12], [205], [206], [207].

### 3.1 Vectorization of retroviruses

Early vectorization of simple retroviruse was initiated from the identification of a genome packaging signal ψ [201], [202], [203], [204]. To obtain replication incompetent virus carrying the gene of interest for transfer, the genome of MoMLV was chosen to separate into two plasmids, namely a transfer plasmid and a packaging/helper plasmid [20], [202], [203], [204]. The ψ and intact LTR that required for the transgene packaging into the virion and following host genomic integration were included into transfer plasmid, and the *gag*, *pol*, and *env* genes that are essential for virion assembly were contained in helper plasmid. Theoretically, due to the lack of ψ in helper construct, the transcribed viral DNA of *gag*, *pol*, and *env* prevented from being incorporated into the virion to produce replication competent virus (RCV). However, ψ could be relocated from transfer plasmid to helper plasmid through just a single recombination event, which bearing the significant likelihood of RCV generation. The concept of introducing more obstacles for reaching the linear alignment of LTR, ψ, *gag*, *pol*, and *env* led to the advent of three-plasmid γ-RV system, in which helper plasmid was further split into two: the *gag* and *pol* are remained in packaging plasmid and *env* transferred to third envelope plasmid. The resulting vector system showed comparable effectiveness at integration and sustaining transgene expression [12], [208], meanwhile basically eliminating RCV formation [19], [198], [209], [210], [211].

**Fig. 3:**
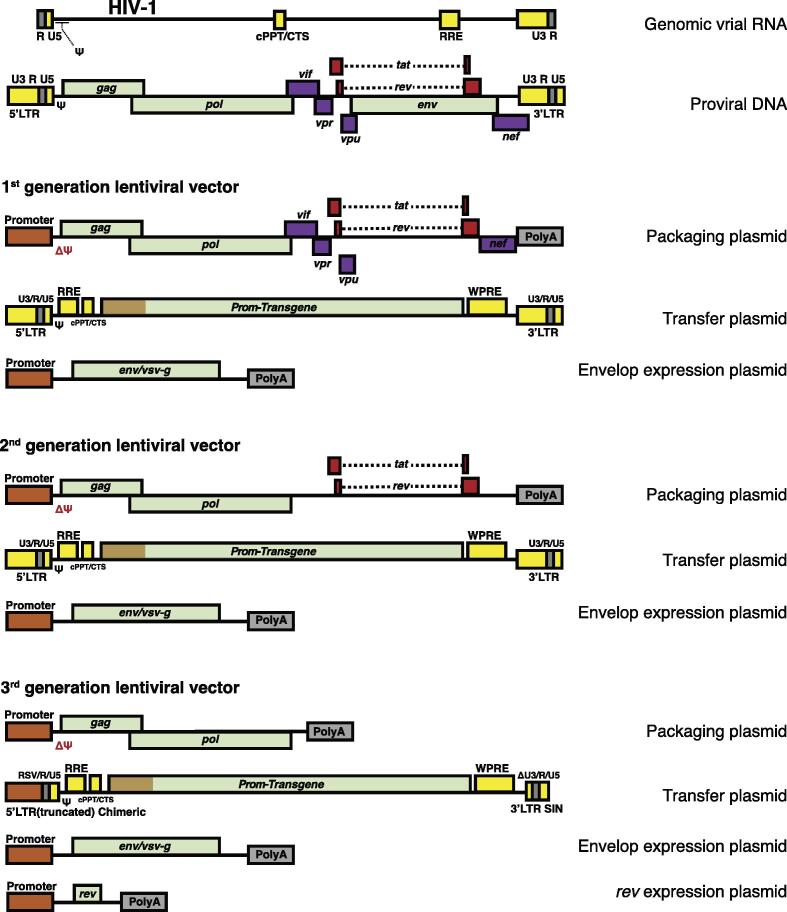
Schematic of HIV-1 and the evolution of lentiviral packaging systems. Lentiviral system derived from HIV-1 has been advanced in iterative cycles toward safety improvement and reduction in the viral replicative competency. During this process, the HIV-specific accessory genes that contributing to pathogenesis but nonessential for viral assembly are removed, then *tat* gene is also removed when the wild type 5′ LTR has been modified. Pseudotype *env* gene from vesicular stomatitis virus glycoprotein is applied to improve stability and increase tropism. RRE: Rev responsive element; cPPT/CTS: central polypurine tract/central termination sequence; WPRE: woodchuck post-transcriptional regulatory element.

However, the performance of γ-RVs was cell-cycle dependent, incapable of transducing quiescent or terminally differentiated cells [56], [212], [213]. Lentiviral systems were developed to overcome the deficiency of γ-RVs systems. Similarly, lentiviral system has been advanced in iterative cycles toward safety improvement and reduction in the viral replicative competency (shown in the Fig. 3). The 1st generation LVs was three-plasmid system derived from HIV-1, including a ψ-containing transfer plasmid with wild-type 5′ and 3′ LTR, a packaging plasmid containing all viral genes with the exception of the *env* gene, and an envelope plasmid containing pseudotype *env* gene from vesicular stomatitis virus glycoproteins (VSV-G) to improve stability and increase tropism. In the 2nd generation, the HIV-specific accessory genes (*nef*, *vif*, *vpu*, and *vpr*) that contributing to pathogenesis but nonessential for viral assembly were removed. Further on, in the 3rd generation, the wild type 5′ LTR was modified into a chimeric promoter that is no longer tat-transactivation dependent and accordingly, *tat* was removed from the packaging plasmid. Apart from that, the TATA box and transcription factor-binding sites within wild type 3′ LTR were also deleted, creating a SIN packaging system. Additionally, the expression of *rev* is further transferred into the 4th plasmid, resulting four-plasmid packaging system. Indeed, the 3rd generation SIN-LV system utilizes only three HIV-1 genes *gag*, *pol*, and *rev* for production, creating the best safety profile in terms of RCV formation in all retrovirus-based delivery platforms [55], [214].

## 4 Next-generation gene editing tools

The *ex vivo* gene therapy based on the addition of exogenous gene has made remarkable progress during the past four decades, demonstrating promising therapeutic results across a wide spectrum of diseases. However, multiple challenges remain to be addressed in the future, such as high variation of transgene expression, unintended effects on neighboring genes, viral packaging limits, unresolvable dominant mutation effects, and so on. Overcoming these fundamental limitations underlying augmentation strategy requires radically distinct schemes, in particular, such as directly altering nucleic acid base pairs via game-changing gene editing approach [215], [216]. Recent advances in programmable endonucleases technologies, such as ZFNs, TALENs, CRISPR/Cas and even more recently emerged base editing, allowed site-specific modifications in genome scale become possible, strategically transforming the option to harvest genetically modified therapeutic cells for *ex vivo* therapy [217], [218], [219], [220], [221], [222], [223].

Programmable endonucleases create site-specific double-strand breaks (DSBs) that mainly resorted into two endogenous pathways for DNA repair, the error-prone NHEJ pathway and the high-fidelity HDR pathway. NHEJ can effectively produce gene knockout, resulted from NHEJ-induced random insertion or deletion causing frameshift mutation if occurring in the coding region of a gene. For HDR, the DNA cleavage is repaired via homologous recombination with a donor DNA carrying the template of homology sequence surrounding the DSB. For this reason, HDR can be exploited to achieve precise gene modifications as designed, eliminating unexpected randomness associated nonhomologous repairs. Indeed, with the property to recognize and bind at specific genomic sites for correction and modification, gene editing approach theoretically evades the genotoxicities attributed to retroviral semi-random genomic insertion, such as proto-oncogenes ectopic activation, tumor suppressor genes knockout or perturbation of splicing [1]. Each type of programmable endonuclease consists of an enzymatic cleavage domain that is nonspecific and a customizable sequence-specific DNA binding domain. Overview of key biological features of each gene editing system have been extensively reviewed previously and briefly summarized here in Table 3 [215], [224], [225], [226].

**Table 3:** Comparison of three engineered endonucleases-ZFN, TALEN, and CRISPR/Cas9.

	**ZFN**	**TALEN**	**CRISPR/Cas9**
Target sequence	9–18 bp per ZFN monomer	15–20 bp per TALEN monomer	20 bp plus PAM sequence
Mode of recognition	Protein:DNA	Protein:DNA	RNA:DNA
Endonuclease	FokI	FokI	Cas
Delivery	Two ZFNs around the target sequence are required	Two TALENs around the target sequence are required	sgRNA with Cas9
Construction	Zinc finger sequence specifically recognizing 3-nucleotide sequence linked to FokI	Protein sequence specifically recognizing 1-nucleotide sequence linked to FokI	20-nucleotide sgRNA complementary to the target sequence fused to Cas9
Reprogramming efficiency	Relatively low	Relatively low	High (easy to design, fast to synthesize)
Off-target effects	High	Low	Variable

Clinically, according to the clinicaltrials.gov, the registered studies based on gene editing as treatment modality mainly concentrated on *ex vivo* gene therapy and overwhelmingly focused on enginerring HSCs to combat infection, malignancies and globinopathies (indicated in Table 4). Gene editing has shown a rapid increase in the recent years and there is a shift of the selected programmable endonucleases overtime. The first ever registered *ex vivo* genome editing trial was in 2009 (NCT00842634), which applied ZFNs. Between 2009 and 2015, only around 2 trials were registered per year. 2016 marks the beginning of TALENs and CRISPR/Cas9 clinic application when both started to be registered for trials, and since 2016 the number of registered trials has increased substantially, with CRISPR/Cas9 becoming the most selected editing system for trial studies.

**Table 4:** Selected early *ex vivo* gene therapy trials with genome editors.

**Nuclease**	**Target gene and effect**	**Disease**	**Sponsor organization**	**Country**	**NCT number**	**Date posted**
ZFN	*CCR5* knockout	HIV-1	University of Pennsylvania	USA	NCT00842634	2009 Feb
ZFN	*CCR5* knockout	HIV-1	Sangamo Biosciences	USA	NCT01044654	2010 Jan
ZFN	*CCR5* knockout	HIV-1	Sangamo Biosciences	USA	NCT01252641	2010 Dec
ZFN	*CCR5* knockout	HIV-1	Sangamo Biosciences	USA	NCT01543152	2012 Mar
ZFN	*CCR5* knockout	HIV-1	Sangamo Biosciences	USA	NCT02225665	2014 Aug
ZFN	*CCR5* knockout	HIV-1	University of Pennsylvania	USA	NCT02388594	2015 Mar
ZFN	*CCR5* knockout	HIV-1	City of Hope Medical Center	USA	NCT02500849	2015 Jul
ZFN	Disrupt *BCL11A*	β-thalassemia	Sangamo Biosciences	USA	NCT03432364	2018 Feb
ZFN	*CCR5* knockout	HIV-1	University of Pennsylvania	USA	NCT03617198	2018 Aug
ZFN	Disrupt *BCL11A*	SCD	Bioverativ	USA	NCT03653247	2018 Aug
ZFN	*CCR5* knockout	HIV-1	Case Western Reserve University	USA	NCT03666871	2018 Sep
TALEN	*TCRα*, *TCRβ*, *CD52* knockout; Create universal T-cells	Advanced lymphoid malignancy	Institut de Recherches Internationales Servier	UK, USA France	NCT02746952	2016 Apr
TALEN	*TCRα*, *TCRβ*, *CD52* knockout; Create universal T-cells	Refractory B-ALL	Institut de Recherches Internationales Servier	UK, Belgium, France, USA	NCT02808442	2016 Jun
TALEN	*PD-1* and *CD52* knockout	AML	Cellectis S.A.	USA	NCT03190278	2017 Jun
TALEN	*PD-1* and *CD52* knockout	AML	Cellectis S.A.	USA	NCT04106076	2019 Sep
TALEN	*PD-1* and *CD52* knockout	Multiple myeloma	Cellectis S.A.	USA	NCT04142619	2019 Oct
TALEN	*PD-1* and *CD52* knockout	CD22^+^ B cell acute lymphoblastic leukemia	Cellectis S.A.	USA	NCT04150497	2019 Nov
Cas9	*PD-1* knockout	Metastatic non-small cell lung cancer	West China Hospital, Sichuan University	China	NCT02793856	2016 Jun
Cas9	*TCRα*, *TCRβ*, *B2M* knockout; Create universal T-cells	B-cell leukemia	Chinese PLA General Hospital	China	NCT03166878	2017 May
Cas9	*CCR5* knockout	HIV-1	Affiliated Hospital to Academy of Military Medical Sciences	China	NCT03164135	2017 May
Cas9	*TCRα*, *TCRβ*, *PD-1* knockout	Various malignancies	University of Pennsylvania	USA	NCT03399448	2018 Jan
Cas9	Disrupt *BCL11A*	β-thalassemia	CRISPR Therapeutics	UK, Germany	NCT03655678	2018 Aug
Cas9	Disrupt *BCL11A*	SCD	Vertex Pharmaceuticals Incorporated and CRISPR Therapeutics	USA	NCT03745287	2018 Nov
Cas9	*CISH* knockout	Metastatic gastrointestinal epithelial cancer	National Cancer Institute	USA	NCT03538613	2018 May
Cas9	*CISH* knockout	Metastatic gastrointestinal epithelial cancer	Masonic Cancer Center, University of Minnesota	USA	NCT04089891	2019 Sep
TALEN	*TCRα*, *TCRβ*, *CD52* knockout; create universal T cells (15-year follow-up study)	Advanced lymphoid malignancy	Institut de Recherches Internationales Servier	UK, Belgium, France, USA	NCT02735083	2016 Apr
Cas9	Disrupt *BCL11A* (15-year follow-up study)	β-thalassemia and severe SCD	Vertex Pharmaceuticals Incorporated and CRISPR Therapeutics	USA, UK, Germany	NCT04208529	2019 Nov

PD-1: programmed cell death protein 1; CISH: cytokine-induced SH2 protein; B2M: β-2 microglobin.

More specifically, *CCR5* gene has been chosen as the very first target in human genome editing trial. A naturally occurring mutation *CCR5*-Δ32 that causing the loss of protein expression has been found generally harmless, but renders homozygous carriers of the null allele resistant to HIV-1 infection. This phenomenon was leveraged ingeniously by Gero Hütter 15 years ago [227] and contributed to the cure of HIV-1 infection of “Berlin Patient” [228], [229], and later on the “London Patient” [230], [231]. So far, the majority of HIV-1 therapy trials based on *CCR5* knockout focused on the application of ZFNs (indicated in Table 4), and the first human clinical trial applying CRISPR for *CCR5* disruption was registered in 2017 (NCT03164135) followed by two more similar clinical studies documented at the end of 2021 (NCT05144386 NCT05143307).

The first TALENs clinical application was used for generating “off-the-shelf” universal third-party CD19-CAR modified T cells by knocking out CD52, TCRα and TCRβ, and *ex vivo* engineered T cells have been administrated to the patients with advance lymphoid malignancy or refractory B cell acute leukemia since 2016 (NCT02746952, NCT02808442). In 2016, a lung cancer patient from China became the first person in the world to be treated with a CRISPR therapy at the West China Hospital, Sichuan University (NCT02793856). This study evaluated the safety and feasibility of PD-1-edited T cells for the treatment of refractory metastatic non-small cell lung cancer (NSCLC). Briefly, relying on electroporation of Cas9 endonuclease and gRNA plasmid to disrupt the *PDCD1* gene, *ex vivo* engineered autologous T cells were manufactured prior to infusion into the patients with NSCLC. In April 2020, results based on 12 patients from this trial were published [232], suggesting the treatment was safe with acceptable side effects, meantime showing a tendency that the patients with higher levels of edited cells had better recovery and slower disease progression. The median editing efficiency has achieved 20.1% and the median off-target events was about 0.05%.

Another key focus of CRISPR clinical studies is the treatment of β-hemoglobinopathies. The first patient receiving CRISPR/Cas9-modified HSCs for TDT took place in Germany in February 2019 (CTX001/NCT03655678) and a few months later, the first patient with SCD was treated with the same therapy in Nashville, Tennessee in July (CTX001/NCT03745287). For β-hemoglobin disorders, CRISPR is designed mostly based on HBB mutation-independent strategies (shown in Fig. 2), with the insights into the fetal-to-adult hemoglobin switch and a rare naturally occurring condition of hereditary persistence of fetal hemoglobin (HPFH) [94], [96]. Neonates and infants with TDT or SCD are typically asymptomatic when their fetal hemoglobin (HbF/α2γ2) levels remain high, and become symptomatic during the developmental process of switching from γ- to β-globin for the adult hemoglobin (HbA/α2β2) production. On the other hand, the HPFH caused by the mutations in the regulatory region of the *HBG* gene has been found capable of alleviating the symptoms of SCD, due to the sustained expression of γ-globin [233], [234], [235]. Furthermore, hydroxyurea, the clinically used HbF inducer, has been applied to mitigate the severity of TDT and SCD. So that, the genome editing for the treatment of β-hemoglobinopathies has been mainly directed into two approaches: 1) perturbating the expression of *BCL11A* gene which encoding the fetal hemoglobin silencing factor to induce the production of HbF, or 2) introduction of HPFH or HPFH-like mutations in the *HBG* regulatory region or disrupting *HBG*(γ-globin) repressor binding sites. So far, the therapy based on *BCL11A* perturbation mediated by CRISPR/Case9 has entered clinical trials (NCT03655678 and NCT03745287) and the early clinical results are promising, showing normal to near-normal hemoglobin level recovery with 30%∼40% of hemoglobin as HbF, and the only immediate side effects resulted from the administration of chemotherapy [103].

Genome editing as a potential medical intervention is rapidly advancing into the clinic, however, not only it is still relatively in its translational infancy but the process has turned out to be more difficult than initially thought. Most of all, CRISPR/Cas may exhibit wider-spread off-target effects compared with ZFNs and TALENs and the improvements of CRISPR/Cas in terms of precision and specificity will be crucial for its future development.

## 5 Future perspectives

Since the first approved gene transfer human study, the advancement of gene therapy over the past three decades has ultimately led to multiple regulatory approvals. For *ex vivo* based gene therapies that have been authorized for marketing and many more to come in the future, determining the manufacture location for cell products engineering would be one of the most important issues for the long-term progression in the field. There are basically two different models under discussion regarding where it should take place, centralized model versus decentralized approach [236]. In the centralized setting, the cells from the patient/third party are harvested at local hospital, shipped to center facility for genetic manipulation, then the frozen cell product shipped back to the patient’s location, where the administering physician thaws the cell product and reinfuses it to the patient. The quality control of this manufacturing process secures every cellular product is up to a pre-defined standard, but geographical distance will certainly compromise responsiveness and personalization is usually not particularly prioritized in this model. Conversely, decentralization means cell collection and processing *ex vivo* genetic manipulation locally, vaguely resembling clinically conducted blood transfusion and transplantation of HSCs. Flexibility is naturally implied in decentralizing mode, potentially providing better patient-physician engaging cycles. However, standardization, cGMP manufacturing, regulatory surveillance and personnel training represent great challenges. Automation or semi-automation could be a solution to minimize variability, fluctuation and operational bias for decentralization development. Clearly, for such revolutionary medicine, upcoming innovations should balance between clinical benefit, efficacy, safety, cost-saving and therapeutic value, disease severity, availability and accessibility.

Retroviral delivery platform based on γ-RVs and LVs is instrumental in *ex vivo* gene therapy thanks to the capability to efficiently and permanently integrate into the host cell genome, albeit semirandomly. The pattern of integration can be broadly categorized as absolutely silent insertion, insertion that interferring but biologically irrelevant, insertion that is clinically irrelevant, and transformative harmful insertion. γ-RVs preferentially integrate near transcriptional start sites of active genes, resulting in γ-RVs have a higher chance of interfering with gene transcription contrasted to the intronic integration preference seen for LVs [55], [207], [214], [237]. Thereby, it is basically considered that LVs exhibit overall relatively safer genomic integration profile compared with γ-RVs. As semirandom integration may provide a selective clonal growth advantage, profiling insertion sites and clonal fluctuation should be routinely undertaken after infusion of transduced cells. Currently, the application of insertion-site analyses, such as linear amplification-mediated PCR (LAM-PCR), is rather limited to investigational studies or analyzing abnormal clinical findings, which should be improved in the future for the clinical practice.

The gene editing technology that capable of rewriting human genome sequences has become an entirely new treatment modality in the field of gene therapy. In principle, gene editing should be safer than retroviral vector-based gene addition as it eliminating semirandom genomic integration. However, how to predict off-target effects and how DNA DSBs triggering genome rearrangements such as inversion, translocation even deletion, remain to be answered, demanding better in-depth understanding of the molecular networks underline the genotoxicities associated with each type of programmable endonucleases in genom editing. Moreover, several next-generation editing technologies have embarked on improving the precision, accuracy, efficacy and applicability. For instance, prime editing has realized the precise alterations of DNA sequences without double-strand breaks [238], [239], thus evading DNA repairment related activities, and the development of RNA-targeted editing that conferring the temporal and reversible modification may potentially provide better safety and offer more flexible interventions [240], [241], [242]. Epigenome editing technologies may aid post-transient editor operation with the flexibility in tunability and reversibility [243]. The fast-growing versatile gene-editing technology may alter how we think about gene therapy eventually.

At last, gene therapy is considered the most complex “drugs” ever developed [1], capable of providing durable benefits for devastating, sometime incurable human diseases. The pathway was not linear, but fulfilled with iterative bench-to-bedside cycles [244]. Justified optimism, vigorous global investment, scientific consensus should facilitate their clinical translation and commercialization, increasing the applicability of genetic therapies and unfolding their true potentials.

## CRediT authorship contribution statement

**Xiaomo Wu:** Conceptualization, Writing – original draft, Visualization, Investigation, Supervision. **Xiaorong He:** Investigation. **Fahui Liu:** Methodology, Software. **Xiaochang Jiang:** Methodology, Software. **Ping Wang:** Software, Validation. **Jinyan Zhang:** Software, Validation. **Ju Jiang:** Supervision.
